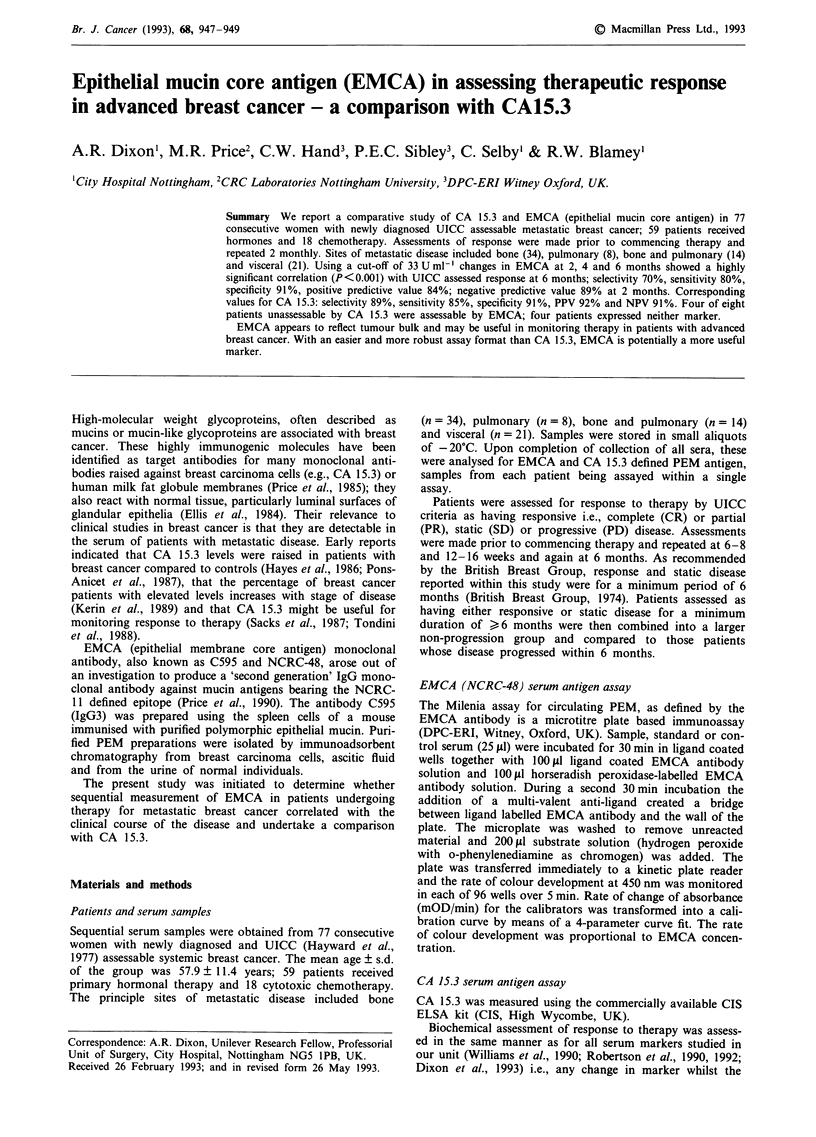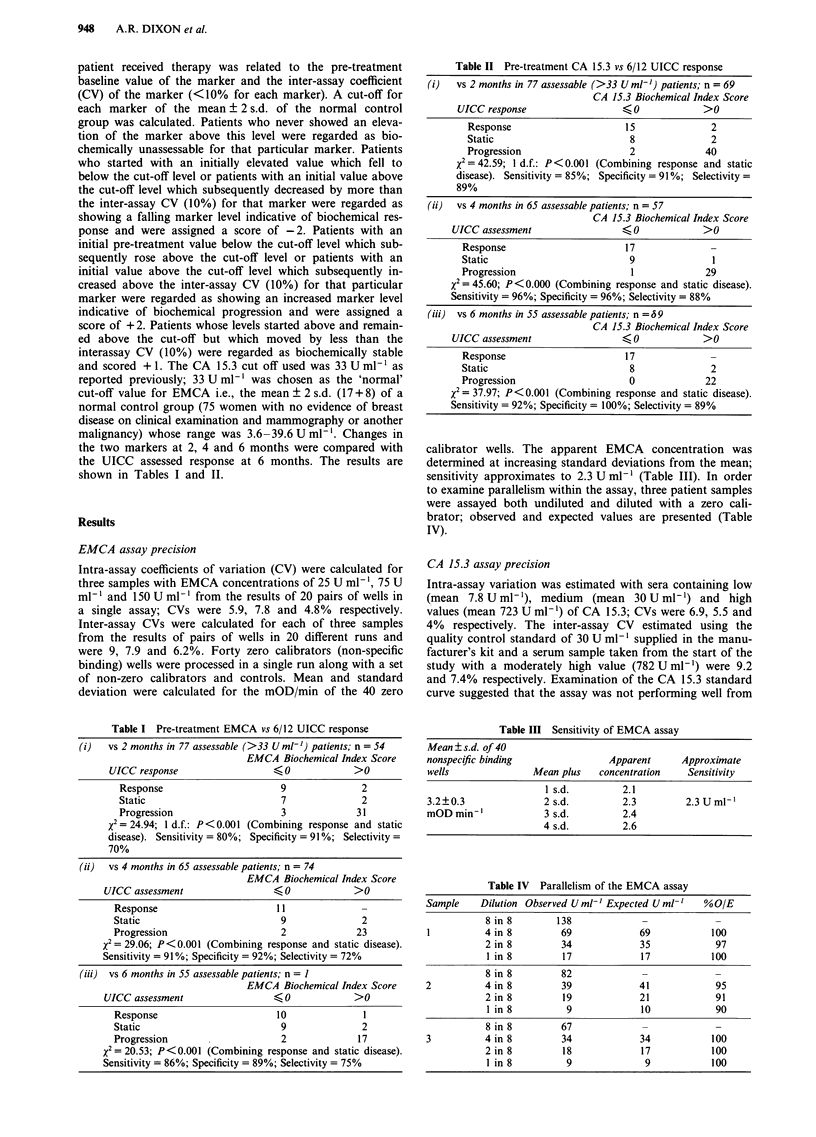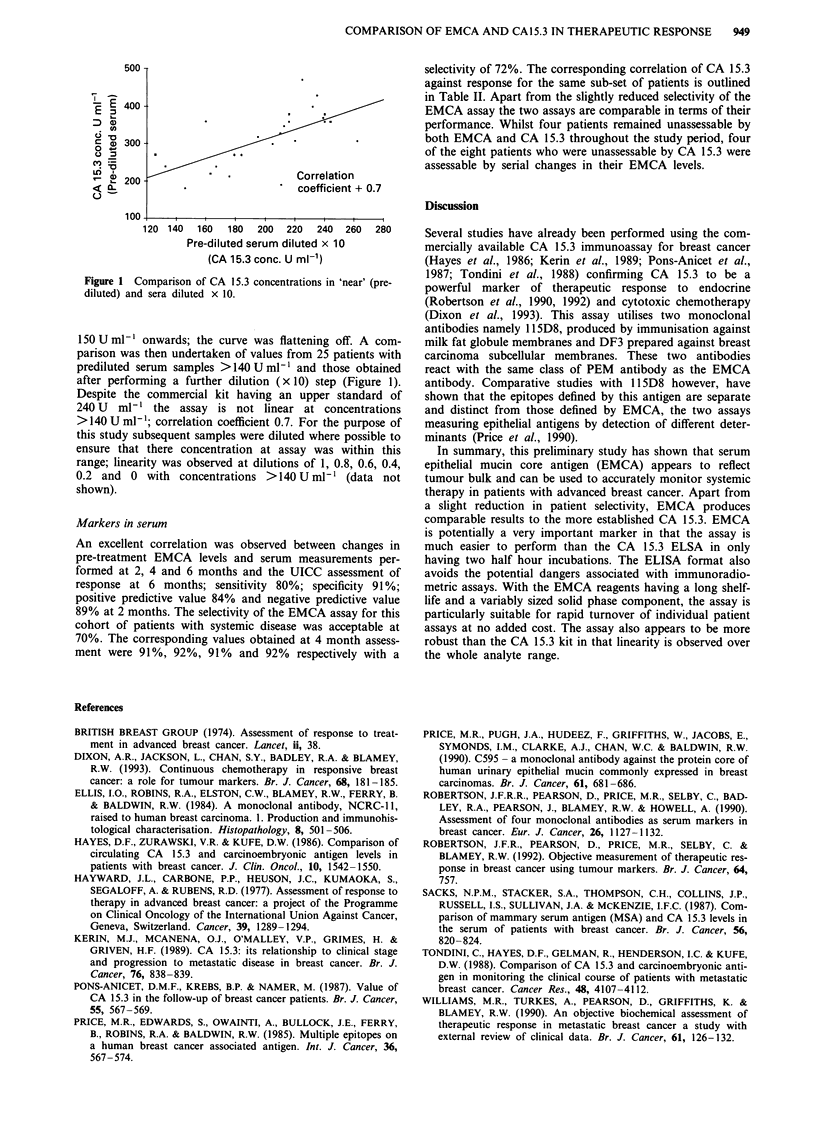# Epithelial mucin core antigen (EMCA) in assessing therapeutic response in advanced breast cancer--a comparison with CA15.3.

**DOI:** 10.1038/bjc.1993.459

**Published:** 1993-11

**Authors:** A. R. Dixon, M. R. Price, C. W. Hand, P. E. Sibley, C. Selby, R. W. Blamey

**Affiliations:** City Hospital Nottingham, UK.

## Abstract

We report a comparative study of CA 15.3 and EMCA (epithelial mucin core antigen) in 77 consecutive women with newly diagnosed UICC assessable metastatic breast cancer, 59 patients received hormones and 18 chemotherapy. Assessments of response were made prior to commencing therapy and repeated 2 monthly. Sites of metastatic disease included bone (34), pulmonary (8), bone and pulmonary (14) and visceral (21). Using a cut-off of 33 U ml-1 changes in EMCA at 2, 4 and 6 months showed a highly significant correlation (P < 0.001) with UICC assessed response at 6 months; selectivity 70%, sensitivity 80%, specificity 91%, positive predictive value 84%; negative predictive value 89% at 2 months. Corresponding values for CA 15.3: selectivity 89%, sensitivity 85%, specificity 91%, PPV 92% and NPV 91%. Four of eight patients unassessable by CA 15.3 were assessable by EMCA; four patients expressed neither marker. EMCA appears to reflect tumour bulk and may be useful in monitoring therapy in patients with advanced breast cancer. With an easier and more robust assay format than CA 15.3, EMCA is potentially a more useful marker.


### Epithelial mucin core antigen (EMCA) in assessing therapeutic response in advanced breast cancer – a comparison with CA15.3

A.R. Dixon[1], M.R. Price[2], C.W. Hand[3], P.E.C. Sibley[3], C. Selby[1] & R.W. Blamey[1]

[1]*City Hospital Nottingham,* [2]*CRC Laboratories Nottingham University,* [3]*DPC-ERI Witney Oxford, UK.*

**Summary** We report a comparative study of CA 15.3 and EMCA (epithelial mucin core antigen) in 77 consecutive women with newly diagnosed UICC assessable metastatic breast cancer; 59 patients received hormones and 18 chemotherapy. Assessments of response were made prior to commencing therapy and repeated 2 monthly. Sites of metastatic disease included bone (34), pulmonary (8), bone and pulmonary (14) and visceral (21). Using a cut-off of 33 U ml$^{-1}$ changes in EMCA at 2, 4 and 6 months showed a highly significant correlation ($P<0.001$) with UICC assessed response at 6 months; selectivity 70%, sensitivity 80%, specificity 91%, positive predictive value 84%; negative predictive value 89% at 2 months. Corresponding values for CA 15.3: selectivity 89%, sensitivity 85%, specificity 91%, PPV 92% and NPV 91%. Four of eight patients unassessable by CA 15.3 were assessable by EMCA; four patients expressed neither marker.

EMCA appears to reflect tumour bulk and may be useful in monitoring therapy in patients with advanced breast cancer. With an easier and more robust assay format than CA 15.3, EMCA is potentially a more useful marker.

High-molecular weight glycoproteins, often described as mucins or mucin-like glycoproteins are associated with breast cancer. These highly immunogenic molecules have been identified as target antibodies for many monoclonal antibodies raised against breast carcinoma cells (e.g., CA 15.3) or human milk fat globule membranes (Price *et al.*, 1985); they also react with normal tissue, particularly luminal surfaces of glandular epithelia (Ellis *et al.*, 1984). Their relevance to clinical studies in breast cancer is that they are detectable in the serum of patients with metastatic disease. Early reports indicated that CA 15.3 levels were raised in patients with breast cancer compared to controls (Hayes *et al.*, 1986; Pons-Anicet *et al.*, 1987), that the percentage of breast cancer patients with elevated levels increases with stage of disease (Kerin *et al.*, 1989) and that CA 15.3 might be useful for monitoring response to therapy (Sacks *et al.*, 1987; Tondini *et al.*, 1988).

EMCA (epithelial membrane core antigen) monoclonal antibody, also known as C595 and NCRC-48, arose out of an investigation to produce a 'second generation' IgG monoclonal antibody against mucin antigens bearing the NCRC-11 defined epitope (Price *et al.*, 1990). The antibody C595 (IgG3) was prepared using the spleen cells of a mouse immunised with purified polymorphic epithelial mucin. Purified PEM preparations were isolated by immunoadsorbent chromatography from breast carcinoma cells, ascitic fluid and from the urine of normal individuals.

The present study was initiated to determine whether sequential measurement of EMCA in patients undergoing therapy for metastatic breast cancer correlated with the clinical course of the disease and undertake a comparison with CA 15.3.

### Materials and methods

### *Patients and serum samples*

Sequential serum samples were obtained from 77 consecutive women with newly diagnosed and UICC (Hayward *et al.*, 1977) assessable systemic breast cancer. The mean age ± s.d. of the group was 57.9 ± 11.4 years; 59 patients received primary hormonal therapy and 18 cytotoxic chemotherapy. The principle sites of metastatic disease included bone ($n = 34$), pulmonary ($n = 8$), bone and pulmonary ($n = 14$) and visceral ($n = 21$). Samples were stored in small aliquots of −20°C. Upon completion of collection of all sera, these were analysed for EMCA and CA 15.3 defined PEM antigen, samples from each patient being assayed within a single assay.

Patients were assessed for response to therapy by UICC criteria as having responsive i.e., complete (CR) or partial (PR), static (SD) or progressive (PD) disease. Assessments were made prior to commencing therapy and repeated at 6–8 and 12–16 weeks and again at 6 months. As recommended by the British Breast Group, response and static disease reported within this study were for a minimum period of 6 months (British Breast Group, 1974). Patients assessed as having either responsive or static disease for a minimum duration of ⩾6 months were then combined into a larger non-progression group and compared to those patients whose disease progressed within 6 months.

### *EMCA (NCRC-48) serum antigen assay*

The Milenia assay for circulating PEM, as defined by the EMCA antibody is a microtitre plate based immunoassay (DPC-ERI, Witney, Oxford, UK). Sample, standard or control serum (25 µl) were incubated for 30 min in ligand coated wells together with 100 µl ligand coated EMCA antibody solution and 100 µl horseradish peroxidase-labelled EMCA antibody solution. During a second 30 min incubation the addition of a multi-valent anti-ligand created a bridge between ligand labelled EMCA antibody and the wall of the plate. The microplate was washed to remove unreacted material and 200 µl substrate solution (hydrogen peroxide with o-phenylenediamine as chromogen) was added. The plate was transferred immediately to a kinetic plate reader and the rate of colour development at 450 nm was monitored in each of 96 wells over 5 min. Rate of change of absorbance (mOD/min) for the calibrators was transformed into a calibration curve by means of a 4-parameter curve fit. The rate of colour development was proportional to EMCA concentration.

### *CA 15.3 serum antigen assay*

CA 15.3 was measured using the commercially available CIS ELSA kit (CIS, High Wycombe, UK).

Biochemical assessment of response to therapy was assessed in the same manner as for all serum markers studied in our unit (Williams *et al.*, 1990; Robertson *et al.*, 1990, 1992; Dixon *et al.*, 1993) i.e., any change in marker whilst the

Correspondence: A.R. Dixon, Unilever Research Fellow, Professorial Unit of Surgery, City Hospital, Nottingham NG5 1PB, UK.
Received 26 February 1993; and in revised form 26 May 1993.

patient received therapy was related to the pre-treatment baseline value of the marker and the inter-assay coefficient (CV) of the marker (<10% for each marker). A cut-off for each marker of the mean ± 2 s.d. of the normal control group was calculated. Patients who never showed an elevation of the marker above this level were regarded as biochemically unassessable for that particular marker. Patients who started with an initially elevated value which fell to below the cut-off level or patients with an initial value above the cut-off level which subsequently decreased by more than the inter-assay CV (10%) for that marker were regarded as showing a falling marker level indicative of biochemical response and were assigned a score of −2. Patients with an initial pre-treatment value below the cut-off level which subsequently rose above the cut-off level or patients with an initial value above the cut-off level which subsequently increased above the inter-assay CV (10%) for that particular marker were regarded as showing an increased marker level indicative of biochemical progression and were assigned a score of +2. Patients whose levels started above and remained above the cut-off but which moved by less than the interassay CV (10%) were regarded as biochemically stable and scored +1. The CA 15.3 cut off used was 33 U $ml^{-1}$ as reported previously; 33 U $ml^{-1}$ was chosen as the 'normal' cut-off value for EMCA i.e., the mean ± 2 s.d. (17+8) of a normal control group (75 women with no evidence of breast disease on clinical examination and mammography or another malignancy) whose range was 3.6–39.6 U $ml^{-1}$. Changes in the two markers at 2, 4 and 6 months were compared with the UICC assessed response at 6 months. The results are shown in Tables I and II.

### Results

### *EMCA assay precision*

Intra-assay coefficients of variation (CV) were calculated for three samples with EMCA concentrations of 25 U $ml^{-1}$, 75 U $ml^{-1}$ and 150 U $ml^{-1}$ from the results of 20 pairs of wells in a single assay; CVs were 5.9, 7.8 and 4.8% respectively. Inter-assay CVs were calculated for each of three samples from the results of pairs of wells in 20 different runs and were 9, 7.9 and 6.2%. Forty zero calibrators (non-specific binding) wells were processed in a single run along with a set of non-zero calibrators and controls. Mean and standard deviation were calculated for the mOD/min of the 40 zero calibrator wells. The apparent EMCA concentration was determined at increasing standard deviations from the mean; sensitivity approximates to 2.3 U $ml^{-1}$ (Table III). In order to examine parallelism within the assay, three patient samples were assayed both undiluted and diluted with a zero calibrator; observed and expected values are presented (Table IV).

### *CA 15.3 assay precision*

Intra-assay variation was estimated with sera containing low (mean 7.8 U $ml^{-1}$), medium (mean 30 U $ml^{-1}$) and high values (mean 723 U $ml^{-1}$) of CA 15.3; CVs were 6.9, 5.5 and 4% respectively. The inter-assay CV estimated using the quality control standard of 30 U $ml^{-1}$ supplied in the manufacturer's kit and a serum sample taken from the start of the study with a moderately high value (782 U $ml^{-1}$) were 9.2 and 7.4% respectively. Examination of the CA 15.3 standard curve suggested that the assay was not performing well from

**Table I** Pre-treatment EMCA *vs* 6/12 UICC response

*(i)* vs *2 months in 77 assessable (>33 U $ml^{-1}$) patients;* n = *54*

| *UICC response* | *EMCA Biochemical Index Score* ⩽*0* | *>0* |
|---|---|---|
| Response | 9 | 2 |
| Static | 7 | 2 |
| Progression | 3 | 31 |

$\chi^2 = 24.94$; 1 d.f.: $P<0.001$ (Combining response and static disease). Sensitivity = 80%; Specificity = 91%; Selectivity = 70%

*(ii)* vs *4 months in 65 assessable patients;* n = *74*

| *UICC assessment* | *EMCA Biochemical Index Score* ⩽*0* | *>0* |
|---|---|---|
| Response | 11 | – |
| Static | 9 | 2 |
| Progression | 2 | 23 |

$\chi^2 = 29.06$; $P<0.001$ (Combining response and static disease). Sensitivity = 91%; Specificity = 92%; Selectivity = 72%

*(iii)* vs *6 months in 55 assessable patients;* n = *1*

| *UICC assessment* | *EMCA Biochemical Index Score* ⩽*0* | *>0* |
|---|---|---|
| Response | 10 | 1 |
| Static | 9 | 2 |
| Progression | 2 | 17 |

$\chi^2 = 20.53$; $P<0.001$ (Combining response and static disease). Sensitivity = 86%; Specificity = 89%; Selectivity = 75%

**Table II** Pre-treatment CA 15.3 *vs* 6/12 UICC response

*(i)* vs *2 months in 77 assessable (>33 U $ml^{-1}$) patients;* n = *69*

| *UICC response* | *CA 15.3 Biochemical Index Score* ⩽*0* | *>0* |
|---|---|---|
| Response | 15 | 2 |
| Static | 8 | 2 |
| Progression | 2 | 40 |

$\chi^2 = 42.59$; 1 d.f.: $P<0.001$ (Combining response and static disease). Sensitivity = 85%; Specificity = 91%; Selectivity = 89%

*(ii)* vs *4 months in 65 assessable patients;* n = *57*

| *UICC assessment* | *CA 15.3 Biochemical Index Score* ⩽*0* | *>0* |
|---|---|---|
| Response | 17 | – |
| Static | 9 | 1 |
| Progression | 1 | 29 |

$\chi^2 = 45.60$; $P<0.000$ (Combining response and static disease). Sensitivity = 96%; Specificity = 96%; Selectivity = 88%

*(iii)* vs *6 months in 55 assessable patients;* n =*δ9*

| *UICC assessment* | *CA 15.3 Biochemical Index Score* ⩽*0* | *>0* |
|---|---|---|
| Response | 17 | – |
| Static | 8 | 2 |
| Progression | 0 | 22 |

$\chi^2 = 37.97$; $P<0.001$ (Combining response and static disease). Sensitivity = 92%; Specificity = 100%; Selectivity = 89%

**Table III** Sensitivity of EMCA assay

| *Mean ± s.d. of 40 nonspecific binding wells* | *Mean plus* | *Apparent concentration* | *Approximate Sensitivity* |
|---|---|---|---|
| | 1 s.d. | 2.1 | |
| 3.2±0.3 | 2 s.d. | 2.3 | 2.3 U $ml^{-1}$ |
| mOD $min^{-1}$ | 3 s.d. | 2.4 | |
| | 4 s.d. | 2.6 | |

**Table IV** Parallelism of the EMCA assay

| *Sample* | *Dilution* | *Observed U $ml^{-1}$* | *Expected U $ml^{-1}$* | *%O/E* |
|---|---|---|---|---|
| | 8 in 8 | 138 | – | – |
| 1 | 4 in 8 | 69 | 69 | 100 |
| | 2 in 8 | 34 | 35 | 97 |
| | 1 in 8 | 17 | 17 | 100 |
| | 8 in 8 | 82 | – | – |
| 2 | 4 in 8 | 39 | 41 | 95 |
| | 2 in 8 | 19 | 21 | 91 |
| | 1 in 8 | 9 | 10 | 90 |
| | 8 in 8 | 67 | – | – |
| 3 | 4 in 8 | 34 | 34 | 100 |
| | 2 in 8 | 18 | 17 | 100 |
| | 1 in 8 | 9 | 9 | 100 |

**Figure 1** Comparison of CA 15.3 concentrations in 'near' (pre-diluted) and sera diluted × 10.

150 U $ml^{-1}$ onwards; the curve was flattening off. A comparison was then undertaken of values from 25 patients with prediluted serum samples >140 U $ml^{-1}$ and those obtained after performing a further dilution (×10) step (Figure 1). Despite the commercial kit having an upper standard of 240 U $ml^{-1}$ the assay is not linear at concentrations >140 U $ml^{-1}$; correlation coefficient 0.7. For the purpose of this study subsequent samples were diluted where possible to ensure that there concentration at assay was within this range; linearity was observed at dilutions of 1, 0.8, 0.6, 0.4, 0.2 and 0 with concentrations >140 U $ml^{-1}$ (data not shown).

*Markers in serum*

An excellent correlation was observed between changes in pre-treatment EMCA levels and serum measurements performed at 2, 4 and 6 months and the UICC assessment of response at 6 months; sensitivity 80%; specificity 91%; positive predictive value 84% and negative predictive value 89% at 2 months. The selectivity of the EMCA assay for this cohort of patients with systemic disease was acceptable at 70%. The corresponding values obtained at 4 month assessment were 91%, 92%, 91% and 92% respectively with a selectivity of 72%. The corresponding correlation of CA 15.3 against response for the same sub-set of patients is outlined in Table II. Apart from the slightly reduced selectivity of the EMCA assay the two assays are comparable in terms of their performance. Whilst four patients remained unassessable by both EMCA and CA 15.3 throughout the study period, four of the eight patients who were unassessable by CA 15.3 were assessable by serial changes in their EMCA levels.

### Discussion

Several studies have already been performed using the commercially available CA 15.3 immunoassay for breast cancer (Hayes *et al.*, 1986; Kerin *et al.*, 1989; Pons-Anicet *et al.*, 1987; Tondini *et al.*, 1988) confirming CA 15.3 to be a powerful marker of therapeutic response to endocrine (Robertson *et al.*, 1990, 1992) and cytotoxic chemotherapy (Dixon *et al.*, 1993). This assay utilises two monoclonal antibodies namely 115D8, produced by immunisation against milk fat globule membranes and DF3 prepared against breast carcinoma subcellular membranes. These two antibodies react with the same class of PEM antibody as the EMCA antibody. Comparative studies with 115D8 however, have shown that the epitopes defined by this antigen are separate and distinct from those defined by EMCA, the two assays measuring epithelial antigens by detection of different determinants (Price *et al.*, 1990).

In summary, this preliminary study has shown that serum epithelial mucin core antigen (EMCA) appears to reflect tumour bulk and can be used to accurately monitor systemic therapy in patients with advanced breast cancer. Apart from a slight reduction in patient selectivity, EMCA produces comparable results to the more established CA 15.3. EMCA is potentially a very important marker in that the assay is much easier to perform than the CA 15.3 ELSA in only having two half hour incubations. The ELISA format also avoids the potential dangers associated with immunoradiometric assays. With the EMCA reagents having a long shelf-life and a variably sized solid phase component, the assay is particularly suitable for rapid turnover of individual patient assays at no added cost. The assay also appears to be more robust than the CA 15.3 kit in that linearity is observed over the whole analyte range.

### References

BRITISH BREAST GROUP (1974). Assessment of response to treatment in advanced breast cancer. *Lancet*, **ii**, 38.

DIXON, A.R., JACKSON, L., CHAN, S.Y., BADLEY, R.A. & BLAMEY, R.W. (1993). Continuous chemotherapy in responsive breast cancer: a role for tumour markers. *Br. J. Cancer*, **68**, 181-185.

ELLIS, I.O., ROBINS, R.A., ELSTON, C.W., BLAMEY, R.W., FERRY, B. & BALDWIN, R.W. (1984). A monoclonal antibody, NCRC-11, raised to human breast carcinoma. 1. Production and immunohistological characterisation. *Histopathology*, **8**, 501-506.

HAYES, D.F., ZURAWSKI, V.R. & KUFE, D.W. (1986). Comparison of circulating CA 15.3 and carcinoembryonic antigen levels in patients with breast cancer. *J. Clin. Oncol.*, **10**, 1542-1550.

HAYWARD, J.L., CARBONE, P.P., HEUSON, J.C., KUMAOKA, S., SEGALOFF, A. & RUBENS, R.D. (1977). Assessment of response to therapy in advanced breast cancer: a project of the Programme on Clinical Oncology of the International Union Against Cancer, Geneva, Switzerland. *Cancer*, **39**, 1289-1294.

KERIN, M.J., MCANENA, O.J., O'MALLEY, V.P., GRIMES, H. & GRIVEN, H.F. (1989). CA 15.3: its relationship to clinical stage and progression to metastatic disease in breast cancer. *Br. J. Cancer*, **76**, 838-839.

PONS-ANICET, D.M.F., KREBS, B.P. & NAMER, M. (1987). Value of CA 15.3 in the follow-up of breast cancer patients. *Br. J. Cancer*, **55**, 567-569.

PRICE, M.R., EDWARDS, S., OWAINTI, A., BULLOCK, J.E., FERRY, B., ROBINS, R.A. & BALDWIN, R.W. (1985). Multiple epitopes on a human breast cancer associated antigen. *Int. J. Cancer*, **36**, 567-574.

PRICE, M.R., PUGH, J.A., HUDEEZ, F., GRIFFITHS, W., JACOBS, E., SYMONDS, I.M., CLARKE, A.J., CHAN, W.C. & BALDWIN, R.W. (1990). C595 - a monoclonal antibody against the protein core of human urinary epithelial mucin commonly expressed in breast carcinomas. *Br. J. Cancer*, **61**, 681-686.

ROBERTSON, J.F.R.R., PEARSON, D., PRICE, M.R., SELBY, C., BADLEY, R.A., PEARSON, J., BLAMEY, R.W. & HOWELL, A. (1990). Assessment of four monoclonal antibodies as serum markers in breast cancer. *Eur. J. Cancer*, **26**, 1127-1132.

ROBERTSON, J.F.R., PEARSON, D., PRICE, M.R., SELBY, C. & BLAMEY, R.W. (1992). Objective measurement of therapeutic response in breast cancer using tumour markers. *Br. J. Cancer*, **64**, 757.

SACKS, N.P.M., STACKER, S.A., THOMPSON, C.H., COLLINS, J.P., RUSSELL, I.S., SULLIVAN, J.A. & MCKENZIE, I.F.C. (1987). Comparison of mammary serum antigen (MSA) and CA 15.3 levels in the serum of patients with breast cancer. *Br. J. Cancer*, **56**, 820-824.

TONDINI, C., HAYES, D.F., GELMAN, R., HENDERSON, I.C. & KUFE, D.W. (1988). Comparison of CA 15.3 and carcinoembryonic antigen in monitoring the clinical course of patients with metastatic breast cancer. *Cancer Res.*, **48**, 4107-4112.

WILLIAMS, M.R., TURKES, A., PEARSON, D., GRIFFITHS, K. & BLAMEY, R.W. (1990). An objective biochemical assessment of therapeutic response in metastatic breast cancer a study with external review of clinical data. *Br. J. Cancer*, **61**, 126-132.